# Study of Impact Characteristics of ZrO_2_ Ceramic Composite Projectiles on Ceramic Composite Armor

**DOI:** 10.3390/ma15041519

**Published:** 2022-02-17

**Authors:** Weizhan Wang, Taiyong Zhao, Fangao Meng, Peng Tian, Guanglei Li, Zhigang Chen

**Affiliations:** 1 National Defense Key Discipline Laboratory of Underground Target Damage Technology, North University of China, Taiyuan 030051, China; zs_991109@163.com (T.Z.); 19940131@nuc.edu.cn (Z.C.); 2 Shandong North Binhai Machinery Co., Ltd., Zibo 255000, China; 18235148123@163.com (F.M.); tianpeng2017@sina.com (P.T.); liguanglei88916@163.com (G.L.)

**Keywords:** ceramic composite projectiles, ceramic projectile tip, penetration, pre-damage

## Abstract

Exploring new armor-piercing materials is crucial for improving the penetrative ability of projectiles. Based on the process of in situ solidification injection molding through ceramic dispersant hydrolytic degradation, a ZrO_2_ ceramic material suitable for use as the tip of a 12.7 mm kinetic energy (KE) projectile was prepared. The ZrO_2_ ceramic tip can be matched with the metal core of a conventional projectile to form a ceramic composite projectile, increasing the damage to the Al_2_O_3_ ceramic composite armor. Specifically, the ZrO_2_ ceramic tip can increase the impact load on the Al_2_O_3_ ceramic panel, prolonging the pre-damage phase and reducing the stable penetration phase, shortening the mass erosion time of the metal core compared with a 12.7 mm metal KE projectile tip. The ceramic composite projectile with the ZrO_2_ ceramic tip has a lower critical penetration velocity than a 12.7 mm metal KE projectile for Al_2_O_3_ ceramic composite armor. Furthermore, the residual velocity, residual length, and residual mass of the metal core of the ceramic composite projectile that penetrated the Al_2_O_3_ ceramic composite armor are greater than those of a 12.7 mm metal KE projectile.

## 1. Introduction

Ceramics are advanced and modern materials, with desirable physical and mechanical properties, such as high hardness, low density, high compressive strength, high speed of sound, wear resistance, corrosion resistance, and heat resistance. Applications of ceramic materials not only include tank armor, but also bulletproof layers of key parts in aircraft, ships, and vehicles. Ceramic materials are also used in body armor, including bulletproof vests and helmets. In practice, ceramic materials can defend against kinetic energy (KE) projectiles and ammunition fragments, and high-performance ceramics have significantly improved the protective armor of many advanced tanks around the world. Ceramics have become an indispensable material for composite armor. However, existing smaller armor-piercing projectiles are usually made from alloy materials. When penetrating ceramic composite armor at high speed, these armor-piercing projectiles are deformed, shattered, fractured, deflected, and crushed owing to the high compressive strength and high hardness of ceramic materials. Thus, the penetration capability of these projectiles is drastically reduced [1,2,3,4,5]. Improvements in armor development have posed new challenges in weapon use, resulting in the search for new materials and structures to enhance combat capabilities. In view of the technical challenges faced by the penetration of conventional ammunition on ceramic armor, such as serious bullet abrasions, relatively high KE loss, and a significant reduction in penetrative effectiveness [6,7,8,9], novel research into penetrative materials has been performed both domestically in China and worldwide. Examples include the impact of ceramic rods and polycrystalline diamond on protected targets [10,11,12], the penetrative effect of ceramic–metal composite projectiles on protected targets [13,14,15], and the impact of Al_2_O_3_ ceramic balls on ceramic composite armor [16]. In particular, research into the penetrative effect of metal rod–ZrO_2_ ceramic ball composites on protected targets is prominent [17], which can cause a large area of damage to the ceramic layer of the ceramic composite armor. At the same time, the development of new ceramic preparation processes has greatly improved the mechanical properties of ceramic materials; for example, post-radiation annealing allows the ceramic amorphous inclusion concentration to be reduced, which leads to a decrease in defects and vacancies in the structure [18,19]. Discharge plasma sintering (SPS), hot press (HP), and hot isostatic pressing (HIP) can improve the mechanical properties of ceramics [20,21,22].

This study utilized the excellent mechanical properties of ZrO_2_ ceramics with high strength and toughness. In situ solidification injection molding through ceramic dispersant hydrolytic degradation was used to prepare high-performance ZrO_2_ ceramic tips, which were matched with the metal cores of conventional projectiles to form ceramic composite projectiles. The ZrO_2_ ceramic tip of the ceramic composite projectiles was used to penetrate the ceramic layer of the ceramic composite armor, and the metal core of the ceramic composite projectiles was used to penetrate the backplate of the ceramic composite armor. This achieved highly efficient armor penetration of the ceramic composite armor and significantly improved the destructive effect on the target protected by ceramic composite armor. The results will have important theoretical and engineering applications in the areas of ammunition combat armor design, armor penetration, and destruction.

## 2. Experimental Tests

### 2.1. Preparation of ZrO_2_ Ceramic Tips

**Figure 1:**
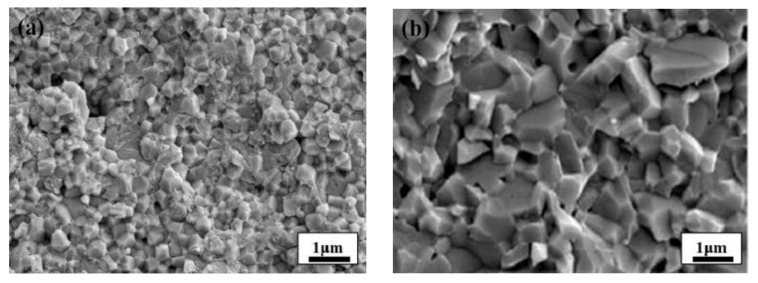
Micrographs [17] of the ceramics: (**a**) Al_2_O_3_ ceramic; (**b**) ZrO_2_ ceramic.

In the initial period, when the projectile hit the ceramic target plate, the number of cracks and fracture time (breakage time of ceramic layer of ceramic composite armor) at the point of impact determined the penetration resistance of the projectile. When the conventional metal projectile hit the ceramic target plate, the number of cracks in the Al_2_O_3_ ceramic target plate was small and the fracture time was short due to insufficient impact force. The projectile was unable to penetrate the target plate owing to the high penetration resistance. As ZrO_2_ ceramic requires higher external energy and a longer time for transgranular fracture (Figure 1a) than Al_2_O_3_ ceramic for intergranular fracture (Figure 1b) [17,23,24], in this paper, a conventional projectile metal tip was designed as a ZrO_2_ ceramic tip with excellent mechanical properties to achieve an efficient impact on an Al_2_O_3_ ceramic target plate.

The experimental zirconia powder was produced by Guangdong Oriental Zirconium Technology Co., Ltd. (Shantou, China) with *d*(0.5) = 0.13 μm and a specific surface area of 8.53 m^2^/g. The dispersant (sodium tripolyphosphate, STPP) was purchased from Sinopharm Chemical Reagent Co. (Shanghai, China) A process of in situ solidification injection molding through ceramic dispersant hydrolytic degradation was used [25].

**Figure 2:**
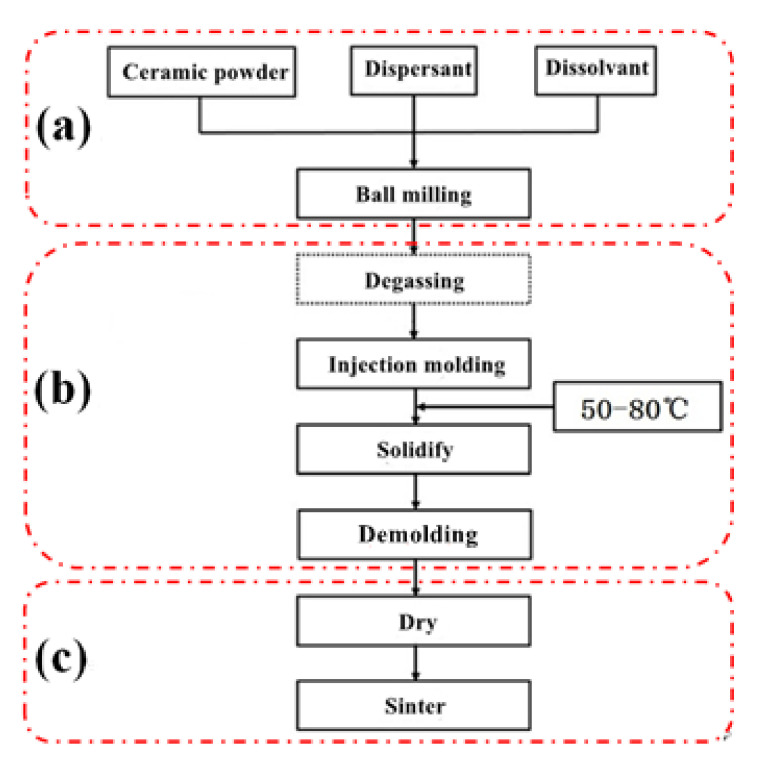
In situ curing injection molding process flow chart for hydrolytic degradation of ceramic dispersants. (**a**) Powder mixing process; (**b**) Molding process; (**c**) Sintering process.

**Figure 3:**
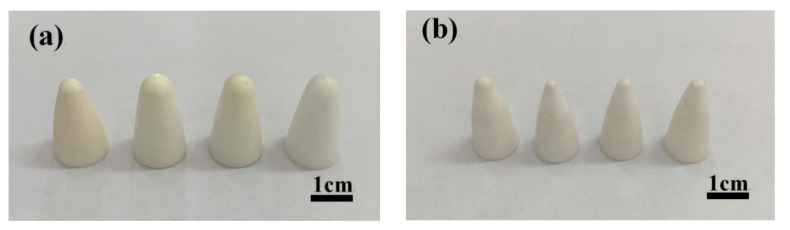
Prepared zirconia bullet tip samples: (**a**) blanks; (**b**) ceramics.

First, zirconia powder, 0.31 wt% dispersant (STPP), deionized water, and grinding balls (the diameter of the grinding balls was 5–20 mm, and the mass ratio to zirconia powder was 1:2) were added to the ball mill cylinder, shaken well, and placed into the ball mill at a speed of 400–500 rpm for 24 h (Figure 2a). Next, the prepared zirconia suspension was poured into a conical flask with a rotor. The conical flask was placed on a magnetic stirrer at a speed of 300 rpm, and the slurry was degassed by a vacuum pump for 30 min, after which the suspension was injected into a non-porous mold coated with a release agent, and the mold was gently shaken to disperse the suspension evenly. The mill was placed into a constant temperature water bath, and the water level of the water bath was adjusted at a level between the liquid level of the suspension and the top surface of the mold. The water bath was heated at a temperature of 50–80 °C until the pH of the suspension was approximately 9 to achieve rapid curing, and the cured blanks were demolded (Figure 2b) and mechanically processed into the dimensions for the ceramic projectile tip structure (Figure 3a). Finally, the processed ceramic projectile tip blanks were dried in the shade for 12 h and then placed in an electric blast drying oven at 70 °C for 12 h. Subsequently, the ceramic projectile tip blanks were placed into a crucible filled with ground alumina powder, and sintered in a chamber furnace at a 5 °C/min rate of heating to 1450 °C, holding at 1450 °C for 2 h, and then cooled at a rate of 5 °C/min (Figure 2c), resulting in a ZrO_2_ ceramic bullet tip sample (Figure 3b).

**Figure 4:**
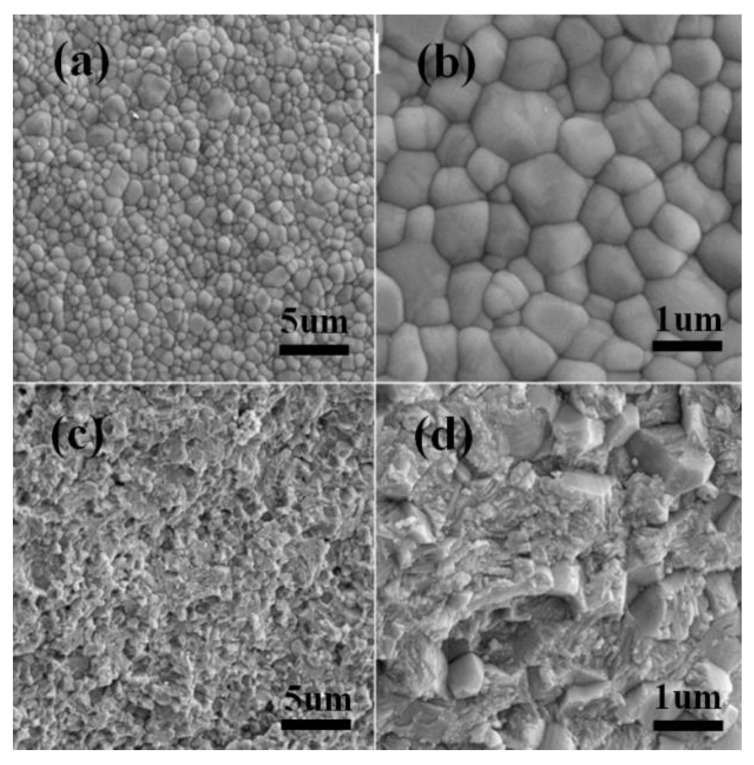
Microstructure of zirconia ceramics: (**a**,**b**) ceramic polished surface after thermal corrosion; (**c**,**d**) fracture surface of ceramics.

Figure 4 shows the zirconia ceramic pattern and microstructure prepared by in situ solidification injection molding through dispersant hydrolytic degradation. A scanning electron microscope, MERLIN VP, from Zeiss, Germany, was used to observe the microscopic morphology of the ceramic sintered samples in a cross-section and polished surface after corrosion. The corrosion process of ZrO_2_ ceramics adopted the method of hot corrosion; that is, polishing is followed by treatment at 1400 °C~1500 °C for 30 min.

Figure 4 shows the microstructure of the corroded samples and fracture surface of zirconia ceramics. The microstructure of the zirconia ceramics was uniform and dense, with no abnormally grown particles and no large pores (Figure 4a,b). Tetragonal zirconia grains were approximately 0.6–1.2 μm in size and exhibited transgranular fractures (Figure 4c,d), the ZrO_2_ crystals in the section were uniformly fractured, and the fracture was relatively flat. From the mechanical parameters of the prepared ZrO_2_ ceramics (Table 1), it is clear that the mechanical properties of zirconia ceramics prepared by the process of in situ solidification injection molding through dispersant hydrolytic degradation process are excellent.

### 2.2. Impact Tests

**Figure 5:**
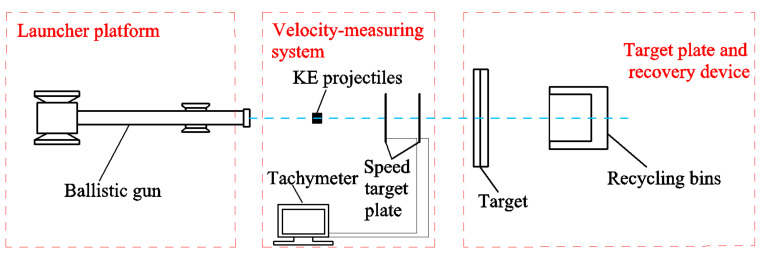
Schematic layout of the test site.

The test was conducted at the laboratory of the National Defense Key Discipline of Underground Target Destruction Technology at the North University of China. The test system consisted of a 12.7 mm ballistic gun firing platform, a laser velocimetry system, and a target box recovery system. The test site was arranged as shown in Figure 5.

**Figure 6:**
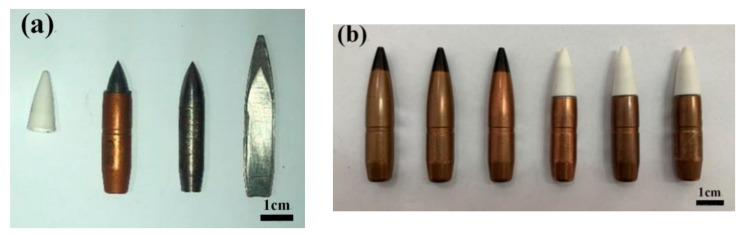
The 12.7 mm test sample: (**a**) ceramic and metal tips; (**b**) metal kinetic energy projectiles (left); ceramic composite projectiles (right).

The test projectiles were Ø12.7 mm × 64.2 mm metal KE and ceramic composite projectiles (Figure 6) with a hardened tool steel (T10) metal core. The tip of the metal KE projectile (armored) was copper-clad steel, the tip of the ceramic composite projectile was ZrO_2_ ceramic, and the ceramic composite target was an 8 mm Al_2_O_3_ ceramic + 2 mm Q235 steel target + 10 mm-thick Kevlar plate composite structure.

## 3. Numerical Simulation

**Figure 7:**
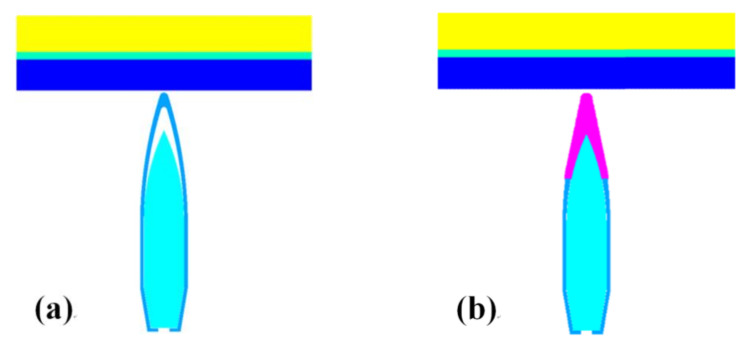
Finite-element models: (**a**) 12.7 mm conventional bullet; (**b**) 12.7 mm ceramic composite bullet.

The simulation was performed using AUTODYN software. To accurately describe the collision process between the 12.7 mm KE projectile and the target plate, the numerical simulation used a 3D calculation model. The ceramic projectile tip and ceramic panel both used the Smoothed Particle Hydrodynamics (SPH) with a particle spacing of 0.1 mm and a total of 66,432 particles, the KE projectile and PE backing plate (The backplate of the ceramic composite armor) both used the Lagrangian algorithm with a grid size of 0.1 mm and a total of 146,326 elements. The finite element type adopts solid hexahedron element, and the contact action between them used the solid–particle contact algorithm. The pressure outflow boundary condition was exerted on the model boundary, which was equivalent to the circumferential stress constraint effect. Figure 7 shows the finite-element model.

The MAT_JOHNSON_HOLMQUIST_CERAMICS material model was used for ZrO_2_ and Al_2_O_3_ ceramics, and the Von Mises material model was used for the KE projectile core, projectile case, and rolled homogeneous armor (RHA) steel material model. The material parameters are listed in Table 2 and Table 3 [17].

## 4. Results

### 4.1. Penetrative Power Analysis

The test results of the 12.7 mm metal KE projectiles and ceramic composite projectiles penetrating ceramic composite armor are listed in Table 4.

**Figure 8:**
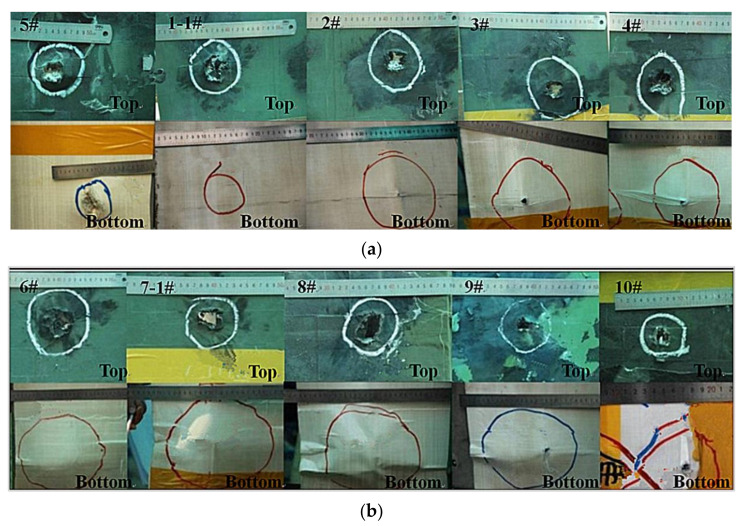
Test results: (**a**) results of ceramic composite projectile penetration; (**b**) results of metal KE projectile penetration.

As indicated in Table 4, compared with the 12.7 mm metal KE projectile (48.3 g), the total mass of the ceramic composite bullet (48.1 g) was smaller, and the critical penetration velocity *V*_50_ of the ceramic composite armor was 471.5 m/s, which was significantly lower than that of the 12.7 mm metal KE projectile (*V*_50_ = 558 m/s). At the same penetration velocity (*V* = 822 m/s), the remaining length and mass of the metal core of the ceramic composite projectile were significantly higher than those of the core of the metal KE projectile. The ceramic composite projectile had a penetrative distinct advantage over the ceramic composite armor. Figure 8 shows the entrance and exit holes of the ceramic composite armor.

The bulge on the ceramic composite armor backplate had a raised cone shape, and the backplate perforation was ductile at low velocities (521–678 m/s), whereas at high velocities (822 m/s), the perforation approximated shear punch plug perforation (Figure 8a,b). The effect of the bulge on the ceramic composite armor backplate under ceramic composite projectile penetration was weaker compared with that of metal KE projectiles (Figure 8a).

**Figure 9:**
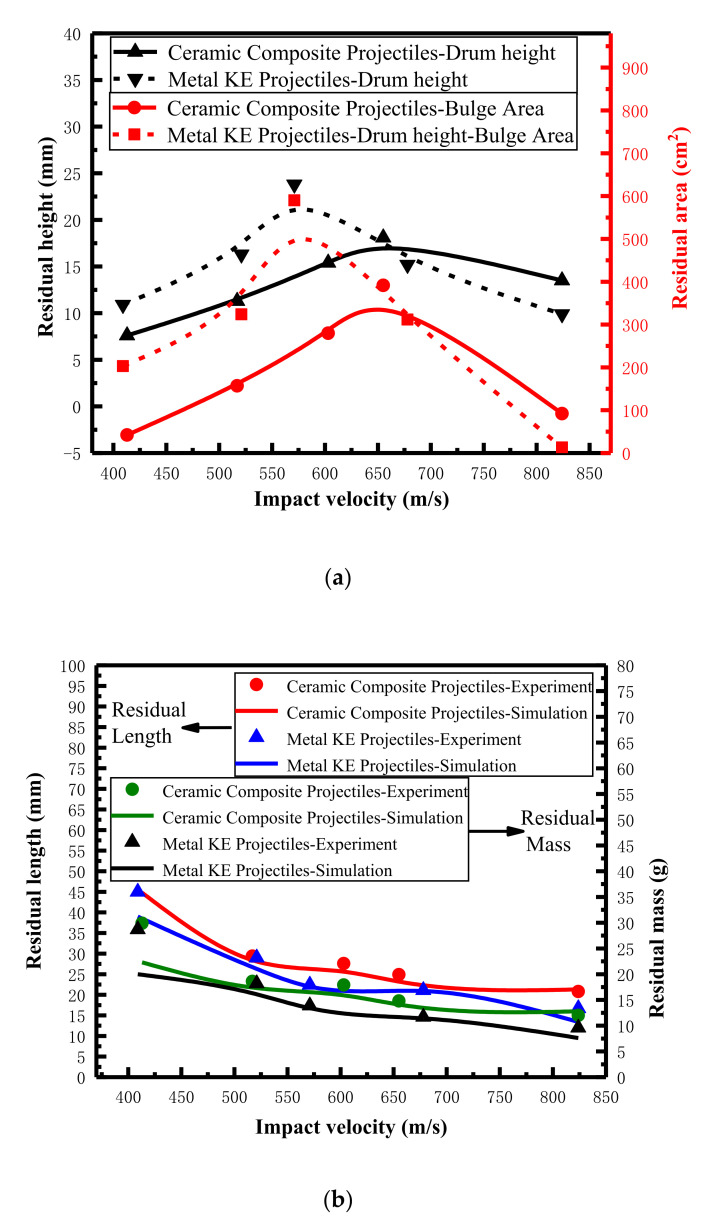
Experimental and numerical simulation results: (**a**) experimental results; (**b**) comparison of experimental and numerical simulation results.

Figure 9 shows the change curves of the area and height of the bulge on the ceramic composite armor backplate and the remaining height and mass of the metal core from the test results.

As shown in Figure 9a, the bulge height and bulge area of the ceramic composite armor backplate increased and then decreased with the increase in penetration velocity. Together with Table 4, the bulge area and bulge height increased with increasing penetration velocity when the core did not penetrate the backplate and decreased with increasing penetration velocity when the core penetrated the target plate. At the same penetration velocity, the backplate bulge area and height from metal KE projectile penetration were larger than those of the ceramic composite projectile. This showed that the deformation of the backplate by the metal KE projectile penetration absorbed more energy and affected the penetration efficiency of the metal KE projectile. The remaining height and mass of the cores of both projectiles in Figure 9b decreased with increasing penetration velocity. The remaining mass and height of the core of the metal KE projectile were smaller than those of the ceramic composite projectile at the same penetration velocity.

**Figure 10:**
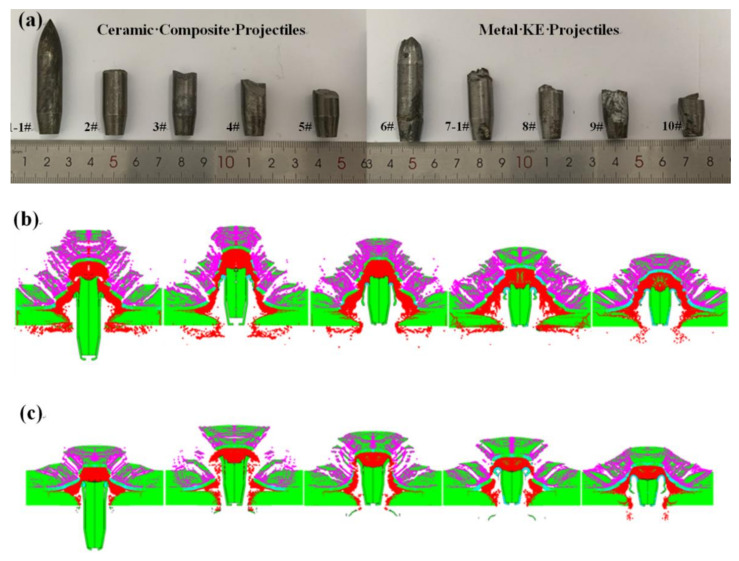
Test results and simulation results: (**a**) test recovered core; (**b**) 12.7 mm ceramic composite core; (**c**) 12.7 mm metal kinetic core.

The remaining length and mass of the metal kinetic energy bullet core in the numerical simulation results showed a decreasing trend with the increase in the intrusion velocity; the deviation of the remaining length and mass of the metal kinetic energy bullet core in the experimental results was small and basically consistent, and the numerical simulation results were consistent with the experimental results (Figure 10)

### 4.2. Analysis of the Penetrative Process

The compressive stress of the ZrO_2_ ceramic tip was different from that of the metal tip during the impact on the ceramic panel [13,14]. The ceramic composite projectile penetrated the ceramic composite armor in two parts, namely, the penetration by the ZrO_2_ ceramic tip and the penetration by the metal core. The impact pressure on the ceramic panel can be solved using [17], and the Hugoniot material parameters for the metal KE projectile tip, ceramic composite projectile tip, and ceramic panel are given in [4,26,27].

**Figure 11:**
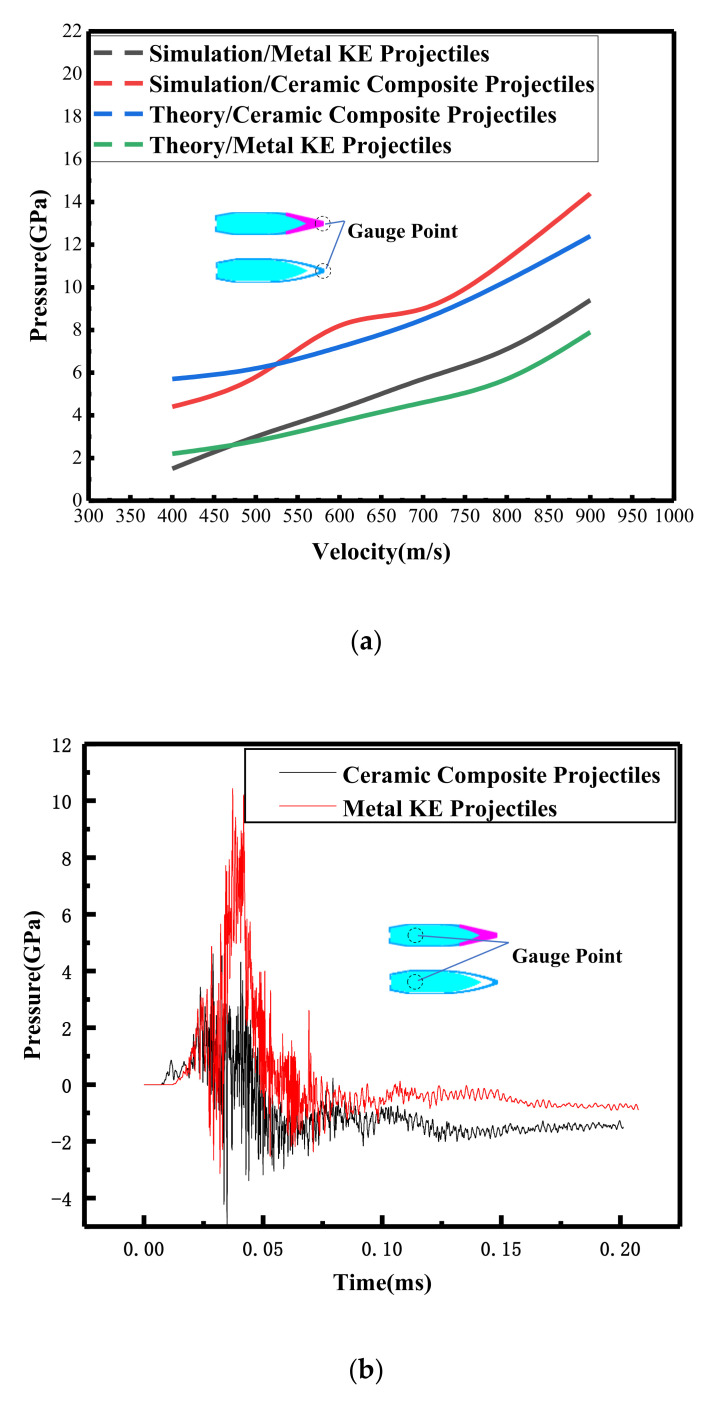
Stress curves for the bullet core micro-element and ceramic panel micro-element: (**a**) micro-element/spring-tip micro-element stress curves for ceramic panels; (**b**) time course curve of micro-element stress in the core for *V* = 822 m/s.

The impact stress of the ceramic tip and the metal KE projectile tip on the ceramic panel increased with the increase in the impact velocity, whereas the impact stress of the ceramic tip on the ceramic panel was greater than that of the metal KE projectile tip. The impact destruction effect of the ceramic tip on the ceramic panel was better than that of the metal KE projectile tip (Figure 11a). Combining the stress time curves of the ceramic composite projectile and the metal KE projectile core micro-element (Figure 11b), it was observed that the stress peak of the ceramic composite bullet core micro-element and the intrusion resistance were lower. The theoretical calculation results were in good agreement with the numerical simulation results.

**Figure 12:**
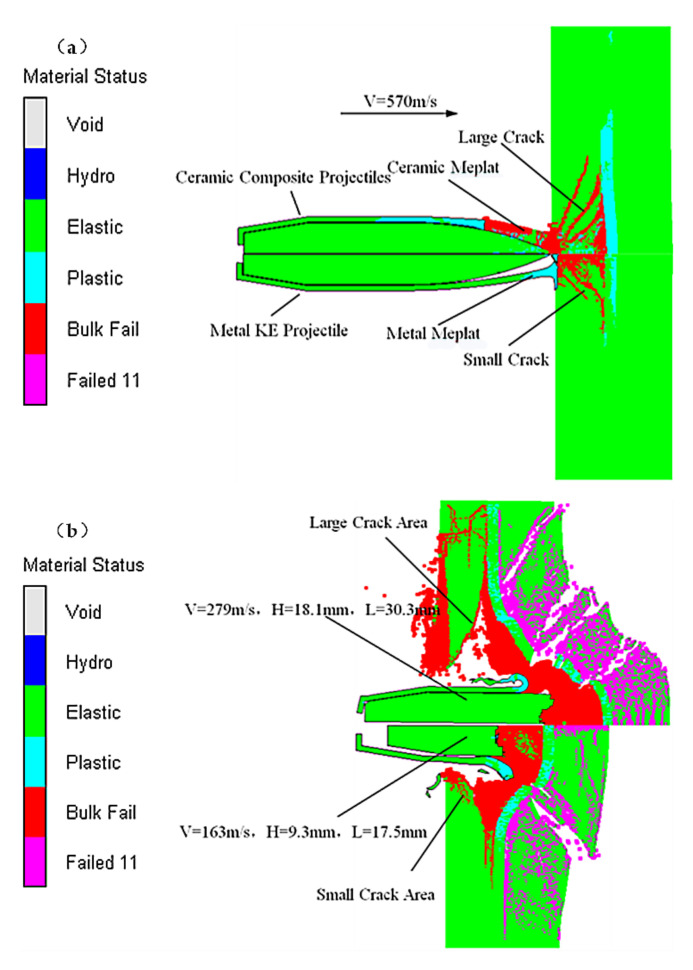

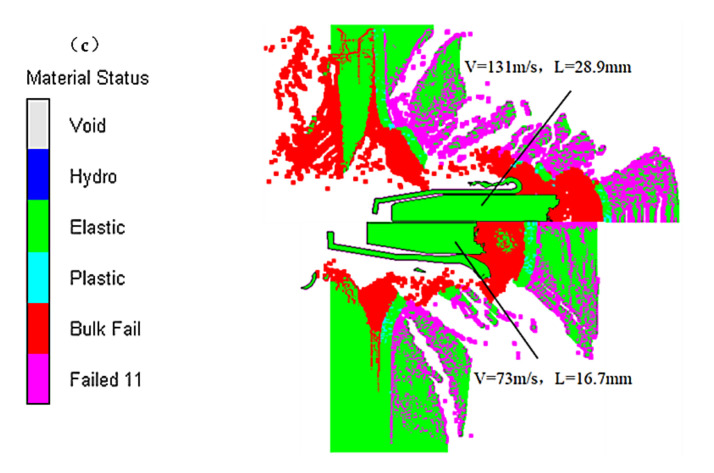
Intrusion process (*V* = 570 m/s): (**a**) pre-damage phase (16 μs); (**b**) stable penetration phase (126 μs); (**c**) penetration or embedding phase (300 μs).

As shown in Figure 12a, when the ceramic projectile tip hit the ceramic panel, the fracture and breakage of the ceramic panel were accompanied by the overall breakage of the ceramic projectile tip, whereas the metal projectile tip was crushed and its mass was slightly deformed and eroded. Together with Figure 11a, the impact stress of the ceramic projectile tip on the ceramic panel was larger, the crack spreading area of the ceramic panel under the impact of the ceramic projectile tip was larger (Figure 11a), and the pre-damage effect of the ceramic composite projectile on the ceramic panel was greater than that of the metal KE projectile. The pre-damage phase for the ceramic composite armor is when the KE projectile tip hits the ceramic panel. After the pre-damage phase, the exposed metal core starts to penetrate the fractured ceramic panel. As the fractured ceramic panel still has a certain structural integrity and target plate resistance under the support of the backing plate, it causes the metal core to break up during penetration, and the degree of breaking depends on the fracture degree of the ceramic panel in the pre-damage phase. This phase is the stable penetration phase. As shown in Figure 12b, the remaining length and penetration depth of the ceramic composite projectile core after fracture were larger than those of the metal KE projectile, which shows that the ceramic composite projectile has an obvious penetration advantage due to its small penetration resistance in the stable penetration stage. When the intrusion velocity of the metal core decreases to the point that the impact stress is not sufficient to cause the metal core to fracture, the metal core and the ceramic fragment as a whole continue to intrude into the backplate until it is embedded or penetrates into the backplate. This stage is the penetration or embedding stage. As shown in Figure 12c, the residual velocity and residual length of the ceramic composite core after penetrating the ceramic composite armor were significantly higher than those of the metal KE projectile.

**Figure 13:**
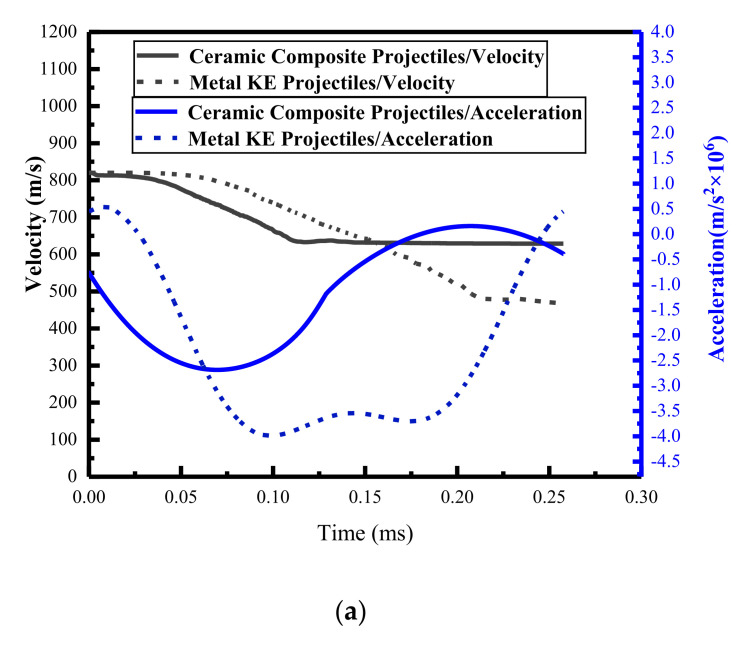

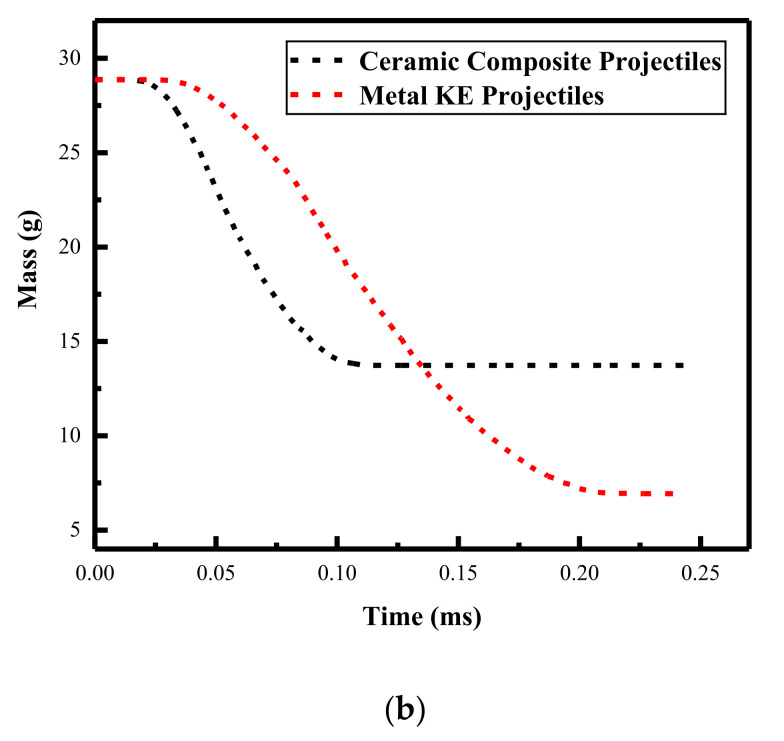
Velocity, acceleration, and mass–time course curves for the metal core: (**a**) velocity/acceleration time course curves; (**b**) three stages of the mass–time curve.

We further developed the analysis of the kinematic characteristics of the metal core (Figure 13) based on the concordance of the numerical simulation with the experimental results and the theoretical calculations (comparison results in Figure 9b, Figure 10 and Figure 11a).

As shown in Figure 13a, during the pre-damage phase (0 < *t* < 0.04 ms), the remaining velocity of the metal KE projectile core was higher than that of the ceramic composite projectile, the acceleration was slower than that of the ceramic composite projectile, and the mass of the metal kinetic projectile core was basically unchanged, whereas the mass of the ceramic composite projectile core gradually eroded (Figure 13b, 0 < *t* < 0.03 ms). This is due to the fact that the metal KE projectile tip has a single-layer cavity shell structure; thus, the core did not come into contact with the ceramic panel during the pre-damage stage, delaying the mass erosion start time. In contrast, in the pre-damage phase of the ceramic composite projectile, the core was in contact with the ceramic tip owing to the dense structure of the ceramic tip, and the stress propagation led to the early fracture of the core. The mass erosion of the core mainly occurred in the stable intrusion stage (Figure 13a, 0.04 ms < *t* < 0.23 ms), the residual velocity of the metal KE projectile core was smaller than that of the ceramic composite projectile, the reverse acceleration was larger than that of the ceramic composite projectile, and the mass erosion loss of the metal KE projectile core was significantly larger than that of the ceramic composite projectile (Figure 13b, 0.04 ms < *t* < 0.23 ms). As the destructive effect of the ceramic composite projectile on the ceramic panel in the pre-damage phase was greater than that of the metal KE projectile, the erosion resistance and mass loss of the metal core in the stable erosion phase were lower.

**Figure 14:**
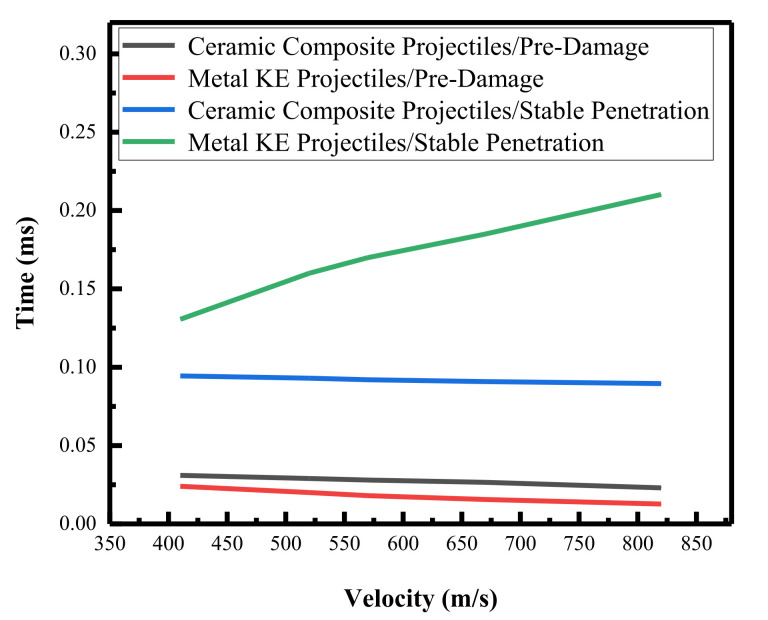
Two phase times versus intrusion velocity.

Figure 14 shows the pre-damage phase and stable penetration phase duration versus impact velocity curves. The pre-damage phase time of the ceramic composite projectile on the ceramic composite armor was greater than that of the metal KE projectile, but the stable penetration time was lower than that of the metal KE projectile. The pre-damage time of both projectiles on the ceramic composite armor decreased with the increase in impact velocity, and the stable penetration time of ceramic composite projectile on ceramic composite armor became shorter with the increase in velocity, whereas for the metal KE projectile, it became longer with the increase in velocity. The analysis showed that the ceramic tip increased the pre-damage time of the ceramic panel, decreased the penetration resistance of the core, and decreased the mass erosion time of the core during the stable penetration phase, effectively increasing the residual velocity and the residual body integrity of the core. This effect became more obvious as the impact velocity increased.

## 5. Conclusions

The ZrO_2_ ceramic tips in this study had high overload performance on impact and could effectively improve the penetrative performance of 12.7 mm KE projectiles against ceramic composite armor. The main advantages of ZrO_2_ ceramic tips over 12.7 mm metal KE projectile tips are as follows.

The mechanical properties of ZrO_2_ ceramic materials prepared by the ceramic dispersant hydrolysis failure in situ solidification injection molding process were found to be superior to those of conventional ZrO_2_ ceramic materials. The ceramic composite projectile with ZrO_2_ ceramic tips could effectively increase the impact stress on the ceramic panel and prolong the pre-damage impact duration, whereas the increase in pre-damage effects on the ceramic panel not only reduced the penetration resistance and micro-element stress of the core, but also shortened the duration of the stable penetration phase of the core, increased the target penetration survival velocity of the core, and reduced the mass loss of the core. In contrast to the 12.7 mm metal KE projectile, the overall performance of the ceramic composite projectile had a lower critical penetration velocity to the ceramic composite armor, and the remaining velocity, remaining length, and mass of the ceramic composite projectile cores penetrating the target plate were greater. The high-performance ZrO_2_ ceramic tip material in this study is of great significance for the structural power design of small caliber armor-piercing ammunition.

## Figures and Tables

**Table 1:** Mechanical properties of ZrO_2_.

Material	Relative Density	Flexural Strength/MPa	Fracture Toughness/MPa/m^2^	Weber Modulus
ZrO_2_	99.0	1100	10.05	18
ZrO_2_ [17]	91.2	850	9.35	-

**Table 2:** Ceramic material parameters (cm-g-us).

Materials	G/GPa	ρ/(g cm^−3^)	A	B	M	N	C	H/GPa
AL_2_O_3_	90.1	3.93	0.9	0.31	0.35	0.6	0.007	4.3
ZrO_2_	155	5.91	1	0.77	1.0	0.38	0.007	6.9

Note: G is the shear modulus; ρ is density; A is the intact strength parameter; B is the fracture strength parameter; M is the strain strength parameter; C is the strain rate strength parameter; M is the pressure fracture strength parameter; H is the elastic limit.

**Table 3:** Material parameters for Q235 steel (cm-g-us).

Material	ρ/(cm^−3^)	E/GPa	PR	Yield/GPa	ETAN	ES
Bullet core	7.86	221	0.28	1.42	0.08	0.4
Projectile case	7.80	207	0.3	0.75	0.03	0.4
Q235	7.83	201	0.3	0.00235	0.01	1.00

Note: ρ is density; E is the modulus of elasticity; PR is Poisson’s ratio; Yield is the yield strength; ETAN is the shear modulus; ES is the mechanical constant coefficient.

**Table 4:** Test results of partial brittle KE projectiles impacting target.

Impact Event#	Projectile Category	Initial Core Mass (g)	Impact Velocity (m/s)	Pierce or Not	Height of Bulge (mm)	Bulge Area (cm^2^)	Final Core Mass (g)
1-1#	Ceramic KE projectiles (mass = 48.1 g, *V*_50_ = 471.5 m/s)	29.69	413	No	7.6	6.7 × 6.3	29.9
1-2#		29.81	434	No	-	-	-
1-3#		29.79	441	No	-	-	-
1-4#		29.82	492	Yes	-	-	-
1-5#		29.77	493	Yes	-	-	-
1-6#		29.69	452	No	-	-	-
2#		29.81	517	Yes	11.3	13.2 × 11.9	18.6
3#		29.73	603	Yes	15.4	17.6 × 15.9	17.9
4#		29.84	655	Yes	18.1	21.4 × 18.3	14.8
5#		29.76	822	Yes	13.5	9.9 × 9.3	12
6#	Metal KE projectiles (mass = 48.3 g, *V*_50_ = 558 m/s)	29.74	409	No	10.9	14.2 × 14.3	28.7
7-1#		29.79	521	No	16.3	18.3 × 17.7	18.2
7-2#		29.79	543	No	-	-	-
7-3#		29.73	579	Yes	-	-	-
7-4#		29.82	536	No	-	-	-
7-5#		29.80	581	Yes	-	-	-
7-6#		29.83	588	Yes	-	-	-
8#		29.79	571	No	23.8	24.9 × 23.7	13.9
9#		29.74	678	Yes	15.2	19.6 × 15.9	11.7
10#		29.83	822	Yes	9.9	11.3 × 10.7	9.6

Note: the critical penetration velocity *V*_50_ is the average velocity of the highest velocity with no penetration and the lowest velocity with penetration. One commonly used standard is to take the average of the velocities recorded for six valid impacts consisting of the three lowest velocities for complete penetration and the three highest velocities for partial or no penetration, provided the spread is not greater than 40 m/s (STANAG 2920).

## Data Availability

Some or all data, models, or code that support the findings of this study are available from the corresponding author upon reasonable request. (Data in Table 4).
